# The Early Publication of the Results of Original Research

**Published:** 1905-03

**Authors:** 


### THE EARLY PUBLICATION OF THE RESULTS OF ORIGINAL RESEARCH.

Of all the various kinds of articles published in medical journals, the most important, all things considered, is the article embodying results of real investigative work, whatever the field occupied may be, in which a real effort has been made to enlarge the bounds of knowledge concerning the problems studied. This is the kind of article that the great American medical public as yet appreciates the least, and we fear that sometimes even medical editors are a little slow in grasping its value. In this country, in particular, medicine needs work of this kind, because we have been so much occupied with organization and with the necessary teaching of old knowledge that there has not been energy and opportunity enough to furnish what would seem to be a creditable share of new knowledge. Consequently, we have become good compilers and facile writers, which at times may be detrimental to critical penetration. It may be taken for granted that no well-educated physician will ever doubt the ultimate practical value of "the knowledge that ripens on the tree of medical science." It takes so long before the new fact, the new point of view, become part and parcel of general medical knowledge that no time should be lost in starting the new fact, or the new idea, on its career of increasing the usefulness of the medical profession. In many quarters doubt, uncertainty and opposition may be aroused by the publication of new work, and this may result in quickening of the investigative activities in many laboratories and clinics. Almost without exception, good new work is sure to prove of direct benefit to other workers in the same and in related fields. A large share of excellent, often epoch-making, investigative work in medicine has been done, and no doubt will continue to be done by comparatively young investigators. To many of them, the satisfaction incident to publicity may be the sole, immediate reward for many hours of patient toil in some obscure corner. Consequently, there is no good reason for unnecessary delay in the publication of the results of investigative work carried to a proper and natural finish. Everything speaks in favor of prompt publication; and in the case of general medical journals, articles of this kind, especially when it concerns problems in preventive and practical medicine, should have the right-of-way over those that do not present original work at first hand.—*Journal American Medical Association.*

